# Developing a Cross-Device Platform for Robotic Systems in Nursing Care: Mixed Methods Feasibility Study

**DOI:** 10.2196/84118

**Published:** 2026-03-19

**Authors:** Pascal Müller, Ruven Veit, Sebastian Hofstetter, Patrick Jahn

**Affiliations:** 1 Health Service Research Working Group, Acute Care, Department of Internal Medicine, Faculty of Medicine, University Medicine Halle (Saale), Martin-Luther-University Halle-Wittenberg, Magdeburger Straße 12, Halle (Saale), 06112, Germany, 49 3455574001; 2 Dorothea-Erxleben-Learning-Centre, Faculty of Medicine, University Medicine Halle (Saale), Martin-Luther-University Halle-Wittenberg, Halle (Saale), Germany

**Keywords:** digital assistive technologies, digital transformation, human–technology interaction, customization, user-centered design, cocreation, intention to use, health care, long-term care, feasibility study

## Abstract

**Background:**

Aging populations and rising chronic illness prevalences are increasing demands for nursing care, while staff shortages threaten care quality. Robotics offer potential support, yet usability, workflow integration, and user acceptance remain major barriers.

**Objective:**

This study aims to develop and evaluate the feasibility, usability, and acceptance of a cross-device platform for controlling robotics in nursing using a participatory, user-centered approach.

**Methods:**

A convergent mixed methods feasibility study was conducted across 4 iterative workshops with 13 nurses from 2 German health care facilities. Quantitative measures included the System Usability Scale (SUS), Technology Usage Inventory (TUI), and Technology-based Experience of Need Satisfaction (TENS-Interface). Qualitative data were collected via think-aloud protocols and focus groups. Data integration supported iterative platform refinement and assessment of usability, acceptance, and satisfaction of psychological needs.

**Results:**

Participants exhibited high curiosity, perceived usefulness, and strong intention to use robotics, with low skepticism. SUS scores indicated acceptable usability. TENS-Interface scores showed increased autonomy and competence following workflow simplification and stepwise interaction logic. Qualitative findings emphasized intuitive control, personalized interventions, centralized management of multiple technologies, integration with documentation systems, and structured training. Triangulation of quantitative and qualitative data confirmed that iterative, user-centered refinements enhanced usability, acceptance, and platform effectiveness.

**Conclusions:**

Cross-device platforms integrating robotics can be successfully developed through participatory, user-centered methods. Technical usability, personalization, workflow integration, and structured training are key for adoption. The study demonstrates that technological barriers, rather than human resistance, are primary constraints to integrating robotics into nursing practice and can be mitigated through iterative co-creation aligned with real-world care contexts.

## Introduction

### Background

Worldwide demographic shifts, including population aging and the increasing prevalence of chronic illness, are driving a growing demand for nursing care, while persistent shortages of qualified nursing professionals threaten the quality and availability of services [1-4]. Robotic systems have therefore been proposed as a promising strategy to support nursing care; however, their implementation in routine practice remains challenging [5]. According to International Organization for Standardization (ISO) 8373:2021 [6], a robotic system is defined as a programmable, actuated mechanism capable of performing physical tasks autonomously or semi-autonomously under the control of a control system. Through the integration of sensors, actuators, and control components, robotic systems can interact with their environment and execute predefined or adaptive actions. This broad definition includes service and assistive robots used in health care settings, such as telepresence robots and mobile manipulators.

In nursing contexts, robotic systems have been shown to support psychosocial and rehabilitative processes, with reported benefits for emotional well-being, cognition, social interaction, and quality of life among older adults [7]. Robots are also used in rehabilitation and physical therapy as well as in psychosocial interventions [8]. Beyond direct patient care, robotic technologies can support organizational processes, including telepresence, virtual consultations, and the training of nursing staff, and can assist with logistical tasks such as transporting medication and materials, disinfection, and procedural support [9]. Despite these potential benefits, most robotic systems remain at early stages of development or testing, and only a limited number have been implemented in routine care settings [10,11]. Moreover, the high financial investment required for acquisition and maintenance continues to be a major barrier to widespread adoption [11].

Previous research indicates that the successful implementation and integration of robotic systems in nursing care depends not only on technical performance but also on user acceptance, contextual implementation processes, and the systematic involvement of end users throughout development and evaluation phases. International reviews and empirical studies consistently demonstrate that sociocultural, organizational, and user-centered factors play a decisive role in facilitating practical adoption, beyond the technical design of robotic systems alone [10,12,13].

Nursing staff generally report a willingness to engage with robotic technologies, driven by perceived benefits such as workload reduction, increased efficiency, and improvements in care quality [14,15]. However, many existing robotic systems do not adequately meet users’ needs with regard to usability, adaptability to individual patient requirements, and integration into established care workflows [7,8,11]. In particular, a lack of intuitive interaction concepts and standardized, cross-device solutions often results in complex and inconsistent operation [8,16,17]. Furthermore, the absence of common standards for the external control of robotic systems remains an unresolved challenge [16,18]. Participatory development approaches, such as cocreation [19] and design-based research [20], have been shown to address these challenges by actively involving end users throughout the design process. Early and continuous stakeholder involvement not only improves system usability and acceptance but may also positively influence care processes and outcomes by aligning technological solutions with user needs and care contexts [21-24]. Nevertheless, robotic systems that provide simple, cross-device, and adaptable interaction concepts and that are co-designed directly with nursing staff remain scarce [24].

### Objective

Robotic systems have the potential to support nursing practice; however, their uptake is limited by insufficient usability and limited adaptability to individual care needs. The Educational Exploration Robot Application Platform (EduXBot) project addresses this gap by developing a simplified and unified interface that enables nursing staff to operate existing robotic systems. The accompanying mixed methods feasibility study pursued two objectives: (1) primary objective: to assess the usability, technology acceptance, and user experience of the EduXBot platform among nursing staff within the framework of an iterative, user-centered design process and (2) secondary objective: to develop a multilevel benefit concept for the platform through an in-depth qualitative evaluation using think-aloud and focus group methods to identify perceived value and areas for further improvement.

Together, these objectives aimed to evaluate the platform’s effectiveness and its potential to support the sustainable integration of robotic systems into nursing practice.

## Methods

### Conceptual Framework

The EduXBot project aims to develop an interaction and control platform that simplifies the use of robotic systems in nursing care. The development process follows a participatory and user-centered approach, explicitly incorporating the expectations, experiences, and practical requirements of end users throughout all phases of the project. This process fosters the development of innovative, practical, and user-centered solutions that are more likely to be adopted in everyday practice [25].

The development process was grounded in user-centered and cocreative principles that combine iterative engagement with end users and structured design cycles. Although Farao et al [26] described a user-centered framework that integrates iterative design and stakeholder engagement, our approach emphasizes active collaboration with end users throughout iterative prototyping, evaluation, and refinement phases consistent with cocreative design practices in participatory technology development.

This methodological approach supports the development of a tailored end product based on nurses’ domain expertise while systematically considering potential implementation consequences [26-28]. Previous research has shown that cocreative and user-centered approaches can improve relevant outcomes and increase the usability and acceptance of technological systems in health care settings [29,30].

**Figure 1.**
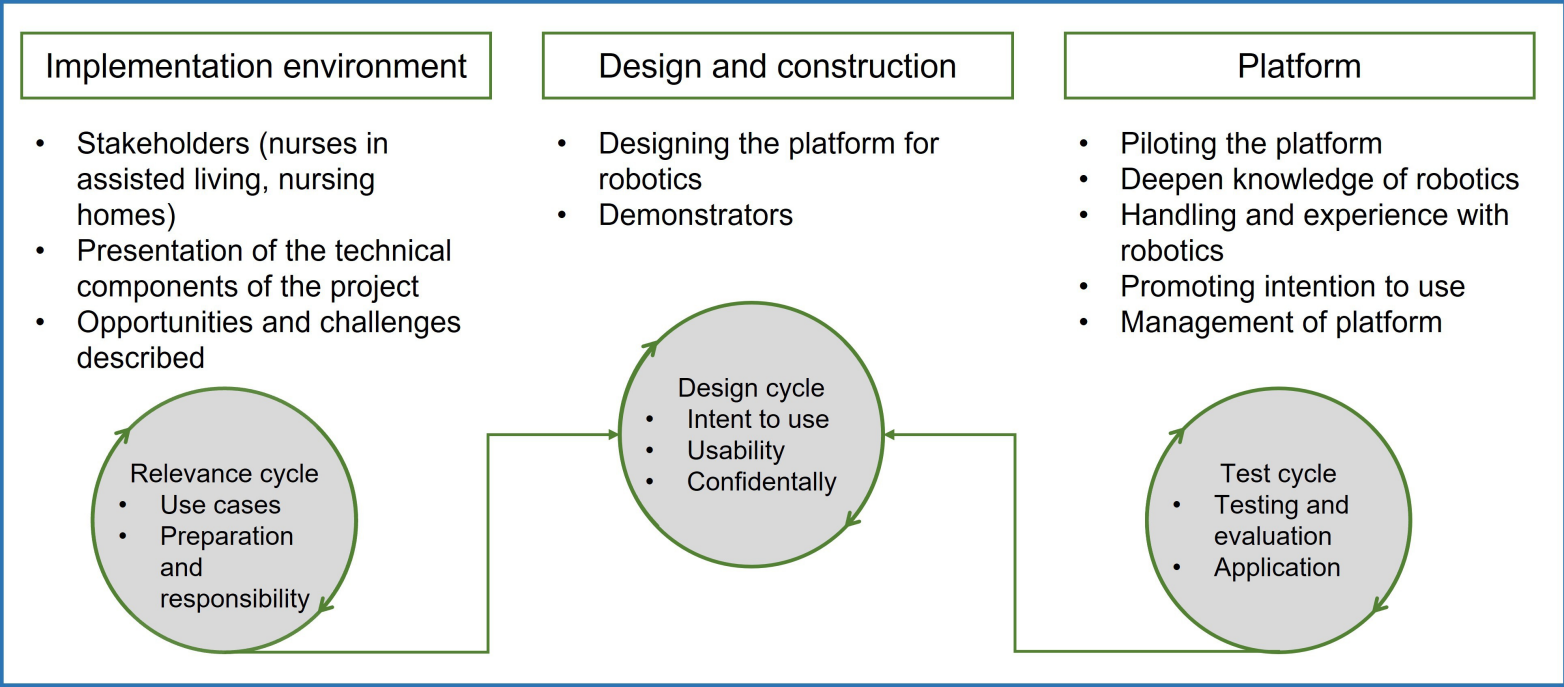
User-centered and cocreative design framework (adapted from Farao et al [26]).

Figure 1 illustrates the integration of relevance, rigor, and design cycles to align nursing practice requirements, scientific evidence, and iterative platform development: the relevance cycle, which captures the realities and needs of end users; the rigor cycle, which draws on the existing scientific knowledge base; and the design cycle, which translates these inputs into the iterative development of the technical product [31-33]. Through this framework, requirements for the robotic systems addressed in the study can be systematically identified and practically operationalized. The close collaboration between researchers and end users further enables active user involvement in product evaluation, thereby supporting the functionality, usability, and overall success of the platform.

Within this collaborative framework, EduXBot does not seek to develop new robotic hardware. Instead, it focuses on providing an extensible interaction and control platform for existing robotic systems. The platform is designed as a flexible and open system architecture that integrates diverse robotic, sensor, and interaction technologies into a unified interface. Its modular design allows nursing staff with varying levels of technological expertise to configure and use the system, thereby supporting a layperson-oriented approach to human-technology interaction. This design was developed in close collaboration with end users to ensure relevance and usability in nursing practice.

### Study Design

An iterative feasibility study accompanied the development of the EduXBot platform. The study was conducted as a convergent mixed methods design in accordance with Creswell and Plano Clark [34], with quantitative and qualitative data collected in parallel and analyzed independently. Quantitative data were used to assess usability, technology acceptance, and the fulfillment of psychological needs, while qualitative data captured nursing staff’s experiences, perceptions, and interaction processes. Following separate analyses, both data strands were integrated to enable a comprehensive interpretation of the findings and to inform the iterative development of the platform. This mixed methods approach aligns with a pragmatic research paradigm, in which methodological choices are driven by the research questions and support a triangulated understanding of the research problem.

Two health care facilities in Germany served as practice partners. Facility 1 was a long-term inpatient care facility. Facility 2 was a regional health center providing acute inpatient care, outpatient services, and long-term inpatient care. Both institutions had previously participated in technology-related research projects and agreed to collaborate in this study. Facility managers were informed about the project and asked to encourage nursing staff to participate.

Participants were recruited using purposive sampling with the aim of maximizing variation in age, gender, and professional experience, thereby capturing a broad range of perspectives relevant to the research topic [35]. The inclusion of nurses from diverse backgrounds enhanced the heterogeneity and informational value of the study sample. Participation was voluntary, and scheduling constraints or other commitments could limit participation or lead to withdrawal during the study.

### Instruments

#### Quantitative Instruments

Surveys were administered at 4 measurement time points (T0-T3) during each development iteration and included the Technology Usage Inventory (TUI), System Usability Scale (SUS), and Technology-based Experience of Need Satisfaction (TENS-Interface). All instruments were administered as self-report questionnaires completed individually by each participant.

The TUI [36] assessed participants’ intention to use robotic systems by measuring technology-related and psychological factors. For this study, the TUI was adapted to address robotic systems in general and was administered once per measurement point. We analyzed 7 subscales: curiosity, fear of technology, interest, skepticism, accessibility, usability, and usefulness. Higher scores indicate stronger expression of the corresponding construct, while lower scores indicate weaker expression. Scale sum scores were converted into stanine values ranging from 1 (well below average) to 9 (well above average).

The SUS [37] measured the usability of each technology integrated into the EduXBot platform. Collecting SUS scores for individual technologies allowed the identification of technology-specific usability issues, providing targeted feedback for iterative development. Medians and IQRs were aggregated to provide a generalizable estimate of the platform’s overall usability, aligning the technology-level measurements with the study’s primary focus on platform-level usability. Scores ≥68 were considered indicative of acceptable usability, supported by adjective-based interpretations from “outstanding” to “very poor.”

The TENS-Interface [38] is a standardized questionnaire that assesses the extent to which a technology interface meets users’ psychological needs (autonomy, competence, relatedness) in human-technology interaction. It was administered from T1 onward, when a prototype was available, and focused specifically on the interaction platform interface.

#### Qualitative Methods

Qualitative data were collected through think-aloud sessions and focus groups. During think-aloud sessions, participants performed predefined tasks for each technology, verbalizing their thoughts and experiences while interacting with the platform [39]. Standardized instructions ensured consistency, enabling systematic observation of interface interaction and usability. Sessions were audio-recorded and transcribed for analysis.

Focus groups were conducted after each workshop [40,41] to refine the platform iteratively. Participants discussed their experiences with the current prototype; evaluated its compatibility with daily care workflows; and identified unmet needs, usability barriers, and desired functionalities. Focus groups were guided by predefined questions, audio-recorded, and transcribed verbatim. Findings were systematically fed back into the design process and informed subsequent platform refinements.

### Data Collection

Prior to the initial measurement (T0), a needs assessment workshop (Table 1) was conducted involving nurses, nursing scientists, and developers. Participants were introduced to existing robotic technologies and collaboratively generated potential application scenarios. Based on practical relevance and technical feasibility, 4 use cases were selected to guide the development of EduXBot prototypes:

1. Conversational partner: implemented via the Double3 telepresence robot, which recognizes residents by voice or face and can respond to personal preferences and topics
2. Telepresence: also using the Double3, enabling remote participants to communicate and engage in activities on site
3. Safe walking partner: implemented via the Go1 4-legged bionic robot, providing monitoring during walks and reporting falls
4. Record/playback of personal events: implemented using the PICO 4 virtual reality (VR) headset, allowing the recording and immersive playback of events such as family birthdays or facility celebrations, to support biographical work

**Table 1.** Overview of the 4 iterative workshops at the 4 measurement time points (T0-T3).

Measurement time point	Thematic focus	Use cases	Technologies	Instruments
T0	Define requirements	Conversational partner Telepresence Record/playback of personal events	Double3 PICO 4	TUI^a^ original questionnaire (pre-post version) SUS^b^ Think-aloud Focus group
T1	Explore concept framework and interaction design	Conversational partner Telepresence Record/playback of personal events Safe walking partner	Double3 PICO 4 Go1	TUI II parallel questionnaire (complete version) SUS TENS-Interface^c^ Think-aloud Focus group
T2	Testing the prototype for interaction design	Conversational partner Telepresence Record/playback of personal events	Double3 PICO 4	TUI II parallel questionnaire (complete version) SUS TENS-Interface Think-aloud
T3	Test simplified step-by-step processes and integrated control	Conversational partner Record/playback of personal events Safe walking partner	Double3 PICO 4 Go1	TUI II parallel questionnaire (complete version) SUS TENS-Interface Think-aloud Focus group

a Technology Usage Inventory.

b System Usability Scale.

c Technology-based Experience of Need Satisfaction.

Data were collected at 4 time points (T0-T3), each corresponding to one iterative development cycle. Workshops followed a standardized structure and integrated evaluation of both the interaction platform and selected technologies. Depending on the development stage, not all technologies nor use cases were presented at every workshop. Table 1 summarizes the technologies and use cases tested at each workshop, along with the applied quantitative and qualitative instruments and thematic focus.

A prototype, defined as a preliminary version of a product or system used to evaluate and refine its key characteristics, design, and functionality prior to final implementation, was used throughout the study [42,43]. Participants interacted in small groups (up to 3 individuals) at station-based setups, rotating between stations to test the prototype and technologies available for that workshop. Usability was assessed at the individual level using the SUS immediately after task completion at each station, ensuring that responses reflected individual perceptions rather than group-based consensus.

After station-based testing, participants completed the TUI and TENS-Interface questionnaires independently and individually, capturing overall intention to use and psychological need satisfaction related to the platform. To minimize potential social influence, participants were instructed to complete all questionnaires independently, and no collective rating nor group agreement was requested at any point during data collection.

Each workshop concluded with a focus group discussion in which participants collectively reflected on experiences, workflow compatibility, usability barriers, and suggested refinements. Insights from think-aloud sessions and focus groups were systematically fed back into the iterative development process and informed subsequent platform and technology adaptations.

### Data Analysis

Quantitative data were analyzed using SPSS version 28 (IBM Corp) [44]. Continuous variables are reported as medians and IQRs, and categorical variables are reported as absolute and relative frequencies. Missing data were handled using pairwise deletion.

Group differences in cumulative SUS and TUI scores at T3 were examined using 1-way ANOVA considering factors such as gender, qualification, and facility. Assumptions of normality and homogeneity of variances were tested, with the Welch correction applied if homogeneity was violated. Post hoc Bonferroni tests were planned but not conducted due to nonsignificant effects. Pearson correlations were calculated to examine relationships between usability (SUS), intention to use (TUI subscales), and psychological need satisfaction (TENS-Interface) at T3. Hierarchical regression analyses assessed predictors of intention to use, with SUS, TENS-Interface, and TUI subscales entered in blocks. Model assumptions (linearity, independence of residuals, homoscedasticity, multicollinearity) were checked prior to interpretation.

Qualitative data were analyzed using inductive content analysis following the method by Kuckartz and Rädiker [45] with MAXQDA version 24.10.0 (VERBI Software) [46]. Transcripts from think-aloud sessions and focus groups were initially coded independently by two researchers (PM, RV). Both methods addressed the same overarching research question—platform design and adaptation of use cases. Coding of the two data sources was therefore integrated in a single schema, enabling triangulation, consistent identification of emerging themes, and comprehensive interpretation across data sources.

Codes were iteratively grouped into subcategories and overarching categories through discussion until consensus was reached. The coding process included systematic comparison across iterations to capture the evolution of requirements, usability challenges, and user perceptions. Intercoder reliability was assessed using Cohen kappa coefficient.

Qualitative data collection was structured according to predefined iterative development cycles rather than aiming for theoretical saturation. The number and timing of think-aloud sessions and focus groups were determined by the 4 development iterations, with each workshop representing 1 iteration of platform refinement. Data collection concluded upon completion of the final iteration, ensuring that all planned prototypes had been systematically evaluated. Emerging themes and usability issues were continuously reviewed after each iteration and directly informed subsequent platform adaptations. This approach was chosen because the study’s objective was to iteratively refine the platform in close collaboration with end users, rather than to achieve saturation of qualitative data.

Mixed methods integration followed a convergent design [34], incorporating both formative and summative integration of quantitative and qualitative data. Formatively, findings from think-aloud sessions and focus groups were continuously fed back into the iterative development process to refine the platform. Summatively, quantitative and qualitative results were triangulated, comparing and integrating findings across data sources to provide a comprehensive understanding of platform usability, technology acceptance, and practical value in nursing workflows. This triangulation allowed complementary perspectives, convergence, and divergence to be identified and interpreted in context.

### Ethical Considerations

The study received ethics approval from the ethics committee of the Medical Faculty of Martin Luther University Halle-Wittenberg (approval 2023‐190, August 31, 2023) and was registered at the German Clinical Trials Register (DRKS00034195). Written informed consent was obtained from all participants, and no financial compensation was provided. Data were collected anonymously to ensure participant privacy and confidentiality.

The methodological rigor of the study was ensured by adhering to the Good Reporting of a Mixed Methods Study (GRAMMS) checklist [47] and the Mixed Methods Appraisal Tool (MMAT) [48]. The comprehensive study protocol has been published elsewhere [49].

## Results

### Participants

Between February 2024 and May 2025, 13 nurses from 2 health care facilities participated in the study. Some nurses participated in multiple iterations, yielding a total of 33 participation units across the 4 development iterations. Participants could not be clearly categorized into core or ad hoc groups due to variable attendance; however, each nurse participated in at least 2 iterations.

Participants’ age, gender, professional qualifications, and experience are summarized in Table 2. Age and professional experience were calculated at the time of each participant’s first iteration to account for staggered entry. Professional experience was defined as years since completion of formal nursing education or training. Median age was 46 (IQR 10.5) years, and the median length of professional experience was 15 (IQR 13.5) years. Most participants held managerial nursing roles, supplemented by support staff and occupational therapists, providing diverse perspectives for assessing the integration of the platform into daily care routines.

**Table 2.** Demographic characteristics of participating caregivers at the 4 measurement time points (T0-T3).

Characteristics		T0 (n=8)	T1 (n=9)	T2 (n=8)	T3 (n=8)
Age (years), mean (SD)		45.6 (9.1)	47.8 (9.3)	50.1 (7.8)	48.3 (9.5)
Gender, n (%)					
Male		1 (12.5)	1 (11)	1 (12.5)	1 (12.5)
Female		7 (87.5)	8 (89)	7 (87.5)	7 (87.5)
Qualification, n (%)					
3-year duration of training		1 (12.5)	1 (11)	0 (0)	1 (12.5)
3-year duration with professional training		2 (25)	1 (11)	1 (12.5)	1 (12.5)
Nursing service management		3 (37.5)	3 (33)	3 (37.5)	3 (37.5)
Studies		1 (12.5)	2 (22)	2 (25)	1 (12.5)
Other qualification		1 (12.5)	2 (22)	2 (25)	2 (25)
Professional experience (years), mean (SD)		13.5 (9)	16.3 (10.8)	16.9 (10.8)	18 (10.5)
Facility, n (%)					
Facility 1		6 (75)	7 (78)	7 (87.5)	6 (75)
Facility 2		2 (25)	2 (22)	1 (12.5)	2 (25)

### Quantitative Evaluation

Participants exhibited higher openness to robotics than the general population, as indicated by above-average TUI stanine scores relative to the normed reference population [36]. Table 3 presents TUI subscale scores including curiosity, fear of technology, interest, usability, usefulness, skepticism, and accessibility. Scores are reported as medians and IQR, with stanine values indicating relative positioning versus the normed reference population. Median intention to use at T0 was 255 out of 300 and remained stable across iterations. High scores were observed for curiosity (median 23 from a possible total of 28) and perceived usefulness (median 22 from a possible total of 28), whereas skepticism was low (median 9 from a possible total of 28). These results indicate general interest in and acceptance of robotics.

**Table 3.** Technology Usage Inventory (TUI) subscale scores at the 4 measurement time points (T0-T3).

TUI subscale	Max score	T0 score		T1 score		T2 score		T3 score	
	Median (IQR)	Stanine	Median (IQR)	Stanine	Median (IQR)	Stanine	Median (IQR)	Stanine
Curiosity	28	22 (6.5)	7	22 (9.75)	7	20 (7.5)	7	23 (9.25)	8
Fear of technology	28	9 (13.5)	5	7 (11)	4	7 (3.75)	4	10 (7.5)	5
Interest	28	18 (12)	6	23 (2)	7	24 (5.5)	7	24 (6.75)	7
Usability	23	18 (4.75)	6	14 (5.5)	4	14 (2.75)	4	17 (5.5)	6
Usefulness	28	22 (4.5)	9	24 (3.5)	9	24 (9)	9	22 (5.5)	9
Skepticism	28	7 (5.75)	2	9 (7.5)	3	10 (4.5)	4	9 (5.75)	3
Accessibility	23	11 (9)	6	11 (4)	6	11 (3.75)	6	11 (3.75)	6
Intention to use	300	255 (69.5)	8	246 (58.5)	7	254 (40.25)	8	262 (46.5)	8

SUS scores were assessed per technology and aggregated to evaluate overall platform usability (Table 4). Median and IQR are reported. Overall usability decreased slightly from “excellent” at T0 to “good” at T3 but remained within the acceptable range. Double3 usability improved over time, whereas Go1 and PICO 4 showed declines, with Go1 falling below the usability threshold at T3. SUS data for Go1 were not available at all measurement points, limiting trend interpretation.

**Table 4.** System Usability Scale (SUS) scores per technology and overall at the 4 measurement time points (T0-T3).

Technology	T0 score, median (IQR)	T1 score, median (IQR)	T2 score, median (IQR)	T3 score, median (IQR)
Double3	68 (22.5)	71 (19.25)	80 (12.5)	75 (—^a^)
PICO 4	85 (12)	85 (30)	69 (15)	70 (27.5)
Go1	—	70 (10)	—	64 (34.38)
Overall	74 (15.7)	75 (18.25)	74 (12.5)	72 (27.81)

a Not applicable.

Table 5 shows caregivers’ perceived satisfaction with autonomy, competence, and relatedness while interacting with the platform. Data were collected using the TENS-Interface from T1 onward. Autonomy and competence increased across iterations, indicating growing confidence and mastery, while relatedness showed minor fluctuations. Overall, the platform interface effectively supported caregivers’ needs, particularly autonomy and competence.

**Table 5.** Technology-based Experience of Need Satisfaction (TENS)–Interface subscale scores at 3 of the 4 measurement time points (T1-T3).

TENS-Interface	T1 score, median (IQR)	T2 score, median (IQR)	T3 score, median (IQR)
Autonomy	18 (3.5)	19 (3.75)	22 (5)
Competence	20 (3.5)	20 (3.5)	23 (3)
Relatedness	16 (5)	18 (1.75)	17 (3.75)

The 1-way ANOVA revealed no significant differences in cumulative SUS nor TUI scores at T3 based on gender, qualification, or facility. Pearson correlations indicated strong associations between SUS and TUI usability (*r*=0.809), usefulness (*r*=0.773), and intention to use (*r*=0.751). Intention to use was highly correlated with perceived usefulness (*r*=0.926). Higher skepticism correlated with lower usability (*r*=−0.856) and usefulness (*r*=−0.736). Greater technology interest correlated with higher curiosity (*r*=0.861). Higher qualification levels were associated with lower technology fear (*r*=−0.800), and more professional experience was associated with lower interest in technology (*r*=−0.766). Competence in handling the interface correlated strongly with autonomy (*r*=0.912) and positively with technology interest (*r*=0.716).

Hierarchical regression analyses indicated that perceived usefulness (*β*=0.908, *P*<0.001) and fear of technology (*β*=0.102, *P*=0.04) were significant predictors of intention to use robotics (*F*_1,6_=1557.926; *P*<0.001; *R*²=0.998; adjusted *R*²=0.997). Multimedia Appendix 1 provides the complete results of the ANOVA, correlation matrices, and regression models.

### Qualitative Evaluation

The qualitative evaluation comprised 7 think-aloud protocols and 3 focus group discussions conducted across 4 iterative workshops. The analysis yielded 36 codes, which were condensed into 13 subcategories and grouped into 4 overarching categories reflecting platform requirements: usability and interaction (category 1), intervention design and implementation (category 2), training and skill development (category 3), and structural prerequisites (category 4). The full coding framework is provided in Multimedia Appendix 2.

Category 1 encompasses user-friendliness and interaction with the platform. Participants emphasized the need for simple and familiar operating concepts, such as gesture- and voice-based input, as well as a streamlined selection process with options for customization. As one caregiver put it, “What’s important is that it’s simple, easy to use, and works in just a few steps*”* (T1, Nurse 3). The platform should also enable centralized management of multiple technologies and provide interfaces to existing systems.

Category 2 focuses on intervention design and implementation. It emphasizes technological autonomy, flexible planning and booking systems, and coordination by nursing staff. Personalization emerged as a central requirement, necessitating adaptation of content to the biographies, mobility levels, and cognitive limitations of care recipients. Participants also highlighted the value of user-specific profiles to support personalization and efficiency: “You would need to create a profile so you can tap on the user who is supposed to use it, and everything that was previously stored for that person is already there*”* (T1, Nurse 3). Interventions were expected to be meaningful and to provide recognizable benefits, thereby supporting individualized care.

The platform was also expected to support training and skill development among care professionals (category 3). This included the provision of structured content, manuals, and step-by-step guides to facilitate independent learning, regardless of prior digital skills. As one participant noted, “If we were to work with it now, we would first need a written, step-by-step guide telling us exactly what we have to do*”* (T1, Nurse 2). In addition, participants emphasized the need for information on care processes and for safe practice environments in which new technologies could be tested.

Structural prerequisites (category 4) describe the framework conditions required for successful implementation. These included stable internet infrastructure, access to technical support, and compliance with privacy and data protection regulations. Financial considerations were also emphasized: “Usability and cost have to match. You need something that really makes sense*”* (T1, Nurse 13). Available resources and the cost-effectiveness of the technology were perceived as decisive factors influencing the platform’s long-term adoption.

Initial intercoder agreement was low (16.28%, κ=0.16), likely due to inconsistent segment boundaries. After refining the coding procedures, including the use of sentence-bounded segments and consolidation of enumerations, agreement increased to 82.19% (κ=0.81), indicating reliable coding for analysis.

### Iterative Development and Final Operating Concept

The platform was refined through 4 iterative workshops, during which qualitative findings were continuously triangulated with quantitative measures (SUS, TUI, and TENS-Interface). This iterative process enabled the identification and resolution of usability and workflow-related issues across successive development stages.

#### Iteration 1: Initial Requirements

In the first iteration, participants defined core requirements for the platform. A tablet-based interface using familiar gestures (eg, tap, swipe, pinch-to-zoom) and voice input was preferred, enabling centralized control of multiple technologies. Integration with existing documentation systems was considered essential to avoid additional workload. Participants also favored concise, context-sensitive guidance: “Sure, absolutely. A brief description. So that the employee knows that I can use it for that purpose” (T0, Nurse 1). Quantitative baseline data indicated acceptable usability and a moderate intention to use robotics.

#### Iteration 2: Conceptual Prototype

**Figure 2.**
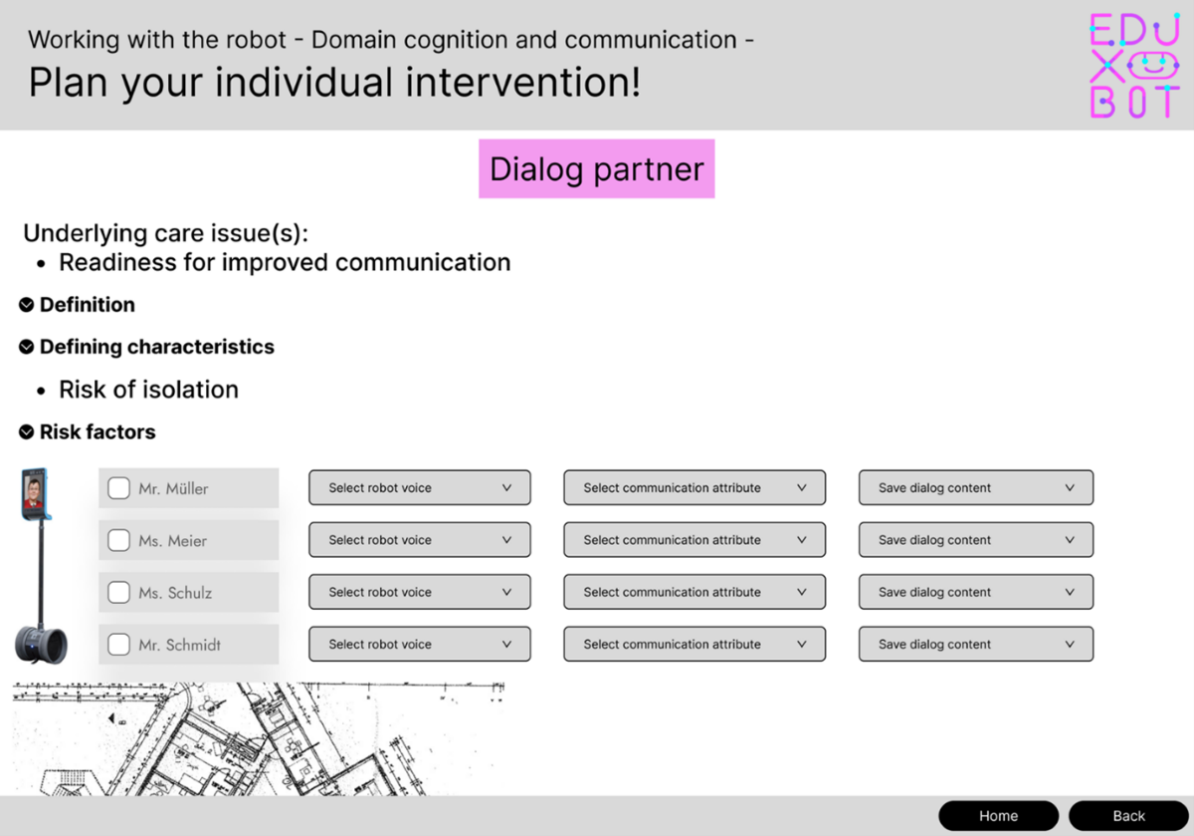
Prototype representation of the Educational Exploration Robot Application Platform (EduXBot) platform (iteration 2).

The second iteration translated these requirements into a visual prototype. The prototype displayed all workflow steps on a single screen, including process information (care problem definitions, risk factors) presented in expandable drop-down menus, personalization options for technological interventions, and information on technology availability and location. Figure 2 shows the platform prototype.

Although participants valued the transparency of having all information readily accessible, extensive vertical scrolling was perceived as inefficient and contrary to the goal of minimizing interaction steps: “So it’s important that it’s simple, easy to use, and can be done in just a few steps” (T1, Nurse 3). The introduction of user profiles reflecting mobility, cognitive abilities, and biographical background to support personalization was positively received. Participants consistently emphasized that interventions should provide a discernible benefit and enable caregivers to actively support residents’ experiences: “What’s the goal? What’s the benefit?” (T1, Nurse 7).

At the same time, participants reiterated the need for written instructions (“So if we were to work with it now, we would first have to get it in writing, know step by step what we have to do*”* [T1, Nurse 2]) and brief training (“You have to practice a little bit, the handling, it takes some getting used to” [T1, Nurse 2]), particularly for more complex technologies.

Quantitative measures indicated stable usability and intention to use. The TENS-Interface showed moderate satisfaction with autonomy, competence, and relatedness. These metrics substantiate the qualitative findings, indicating that usability and acceptance were generally adequate but could benefit from workflow simplification and clearer guidance.

#### Iteration 3: Functional Prototype

In the third iteration, development shifted toward technical implementation. Full functional integration was available only for the PICO 4, while other technologies were simulated. This partial implementation revealed new usability challenges related to system feedback and transparency.

The management of VR content involved storing videos in a backend system, requiring several preparatory steps prior to use. System states were represented graphically using checkmark symbols: A single gray checkmark indicated assignment to a resident, two gray checkmarks indicated availability on the tablet, and two blue checkmarks indicated availability on the VR headset. Participants perceived this symbolic logic as lacking intuitive clarity: “Dots and hooks in blue, gray, and so on. It’s not intuitive*”* (T2, Nurse 7).

Additional confusion arose from synchronization issues between the tablet and the VR headset. Synchronization had to be actively initiated via an eye icon within the video control bar. Participants instead tapped the video preview on the tablet, expecting the VR headset to mirror the same content. Although participants relied on a paper-based manual containing step-by-step instructions, several emphasized that intuitive design should ultimately reduce reliance on such materials.

The absence of explicit safety alerts was identified as a critical shortcoming. During simulated testing of the walking partner use case, participants stressed the need for clear error or event notifications, particularly in the case of falls: “It runs in a specific area, detects falls, and alerts nursing staff via the nurse call system*”* (T2, Nurse 10).

SUS scores for the PICO 4 decreased slightly, indicating usability challenges during partial implementation. In contrast, TENS-Interface scores showed modest improvements in competence and autonomy. This suggests that participants recognized the platform’s potential despite ongoing functional limitations.

#### Iteration 4: Final Operating Concept

**Figure 3.**
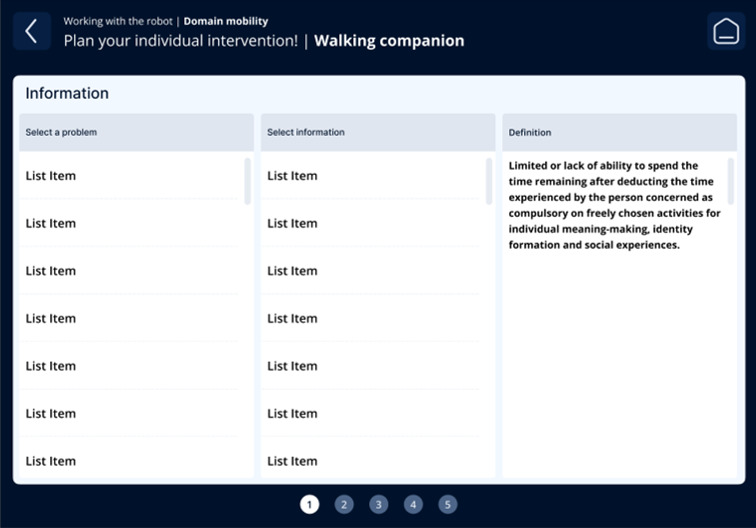
Contextual information on the care problem.

**Figure 4.**
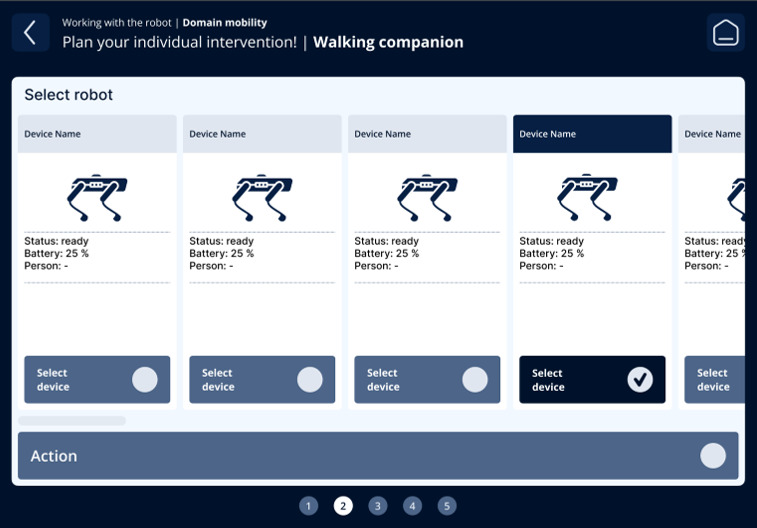
Selection of the appropriate technology.

**Figure 5.**
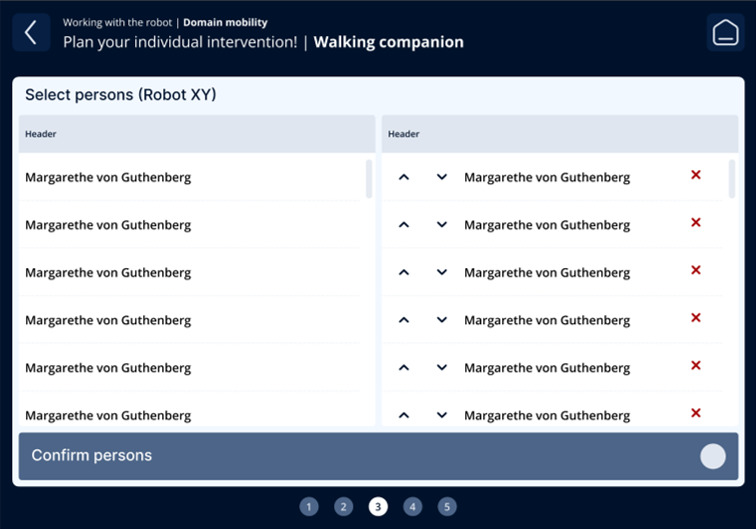
Assignment of the intervention.

**Figure 6.**
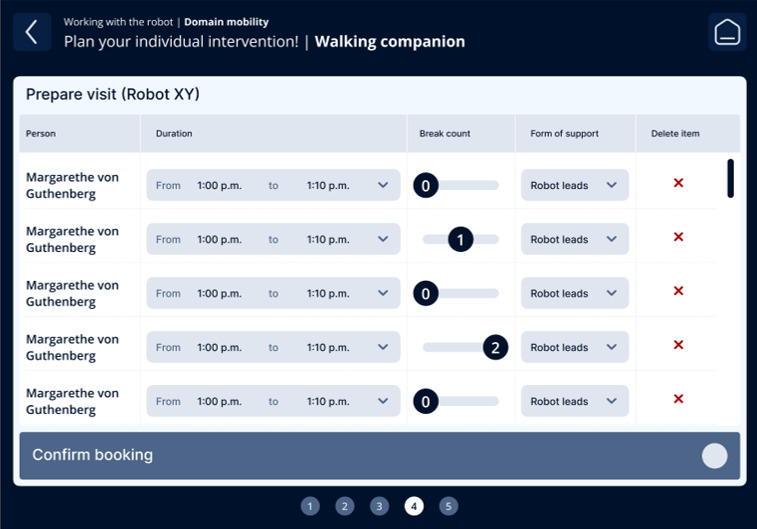
Individualization of intervention parameters and scheduling.

**Figure 7.**
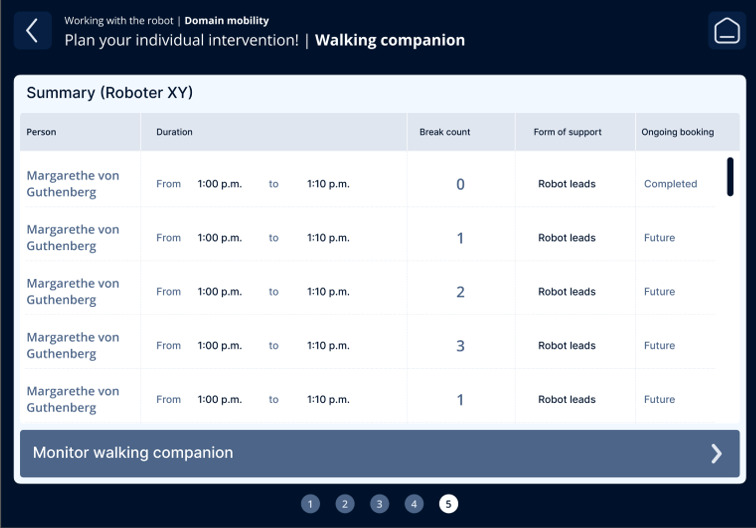
Final overview summarizing all selected settings.

The fourth iteration resulted in a guided, step-by-step workflow aligned with nursing reasoning: (1) contextual information on the care problem clarifying the intended nursing objective of the intervention (Figure 3); (2) selection of the appropriate technology, including system feedback on availability, current usage, and battery status (Figure 4); (3) assignment of the intervention to one or more care recipients (Figure 5); (4) individualization of intervention parameters and scheduling via a booking system (Figure 6); and (5) a final overview summarizing all selected settings with an explicit confirmation step (Figure 7). These figures present the final operating concept.

The execution and monitoring of interventions were also revised. Care professionals retained the ability to enter an ongoing intervention at any time. Preparatory steps, such as file transfers to the tablet or synchronization with the PICO 4, were executed automatically and presented to users via a unified loading indicator. Safety-related events (eg, falls) were implemented as high-priority alerts using prominent pop-up notifications combined with audible warning signals. In addition, a conceptual interface to existing documentation systems was introduced to support routine care documentation, using standardized text modules to demonstrate the transfer of intervention-related information.

Quantitative data indicated stable or improved usability, a high intention to use, and increased satisfaction with autonomy and competence, suggesting that the revised workflow successfully addressed previously identified barriers.

## Discussion

### Integrative Overview

This study examined the feasibility, acceptance, and usage requirements of a cross-device interaction platform for controlling robotic systems in nursing care. Quantitative measures (SUS, TUI, TENS-Interface) and qualitative methods (think-aloud sessions and focus groups) were first analyzed separately and subsequently integrated in a mixed methods approach. The primary research question regarding overall platform feasibility was addressed through usability and interaction measures, while secondary questions concerning workflow integration and personalization were examined through combined TENS-Interface and qualitative feedback. The data were integrated both formatively, to inform iterative platform refinement, and summatively, to evaluate the platform’s overall feasibility and value. This comprehensive approach allowed convergent and divergent findings to be interpreted in context.

### Usability and Interaction

Caregivers demonstrated high levels of curiosity, perceived usefulness, and intention to use robotic technologies, accompanied by low levels of skepticism. Perceived usefulness emerged as the strongest predictor of intention to use robotic systems. These findings indicate that resistance among nursing staff is not the primary barrier to technology adoption. Rather, successful integration depends on whether technological solutions adequately address users’ practical needs.

Although SUS scores revealed usability challenges—particularly for the Go1 robot and the PICO 4 headset—these issues largely reflect the constraints of iterative prototype development rather than fundamental rejection by users. These findings align with the qualitative findings. Early usability challenges were primarily related to complex interaction sequences, unclear system feedback, and inconsistent symbolic representations. Participants emphasized the need for intuitive, gesture- and voice-based input, streamlined workflows, and consistent symbolic representations. Iterative refinements, such as simplified step-by-step procedures and automated preparatory steps, directly addressed these usability challenges. Notably, acceptance and intention to use remained high even when usability temporarily declined.

The triangulated evidence demonstrates that high perceived usability and meaningful interaction design are critical enablers of platform adoption, supporting the primary research question regarding overall platform feasibility. This pattern is consistent with the Technology Acceptance Model, which emphasizes perceived usefulness as a stronger determinant of adoption than ease of use alone [50].

### Personalization and Workflow Integration

Participants consistently emphasized the importance of tangible benefits, including automation of routine processes, integration into existing documentation systems, and the ability to tailor interventions to individual care recipients. Technology skepticism was mitigated when systems were intuitive, workflows were transparent, and benefits were clearly recognizable. These findings align with previous research demonstrating that technologies lacking practical relevance or workflow integration are unlikely to be adopted in nursing care [7,8,11]. The strong emphasis on personalization further supports existing evidence that user-centered and cocreative design approaches enhance acceptance, satisfaction, and care-related outcomes [21-24].

### Training and Implementation Support

Training and implementation support were identified as critical enablers of successful adoption. Participants’ feedback on instructional materials, step-by-step guidance, and protected environments for testing technologies was reflected in increasing TENS-Interface competence scores. Nursing staff reported growing confidence in their ability to use the platform safely and independently. These findings are consistent with Basic Psychological Need Theory, which posits that satisfaction with competence and autonomy fosters motivation and engagement [51]. In line with the Motivation, Engagement, and Thriving in User Experience model, improvements in autonomy and competence were associated with greater willingness to engage with robotic systems [38].

### Structural Preconditions and Organizational Perspective

Although quantitative analyses did not directly capture organizational factors, qualitative findings underscored critical preconditions for successful implementation: stable technical infrastructure, access to technical support, compliance with privacy and data protection regulations, and cost-effectiveness. Participants with managerial responsibilities provided insights into resource allocation and long-term sustainability, emphasizing that organizational and structural factors must be addressed alongside usability and personalization to ensure successful adoption.

### Limitations and Strengths

A major strength of this study is its iterative, user-centered development process, which systematically involved nursing staff through cocreative workshops and repeated prototype evaluations. The integration of qualitative and quantitative data enhanced the depth, credibility, and interpretability of the findings. Adherence to established methodological standards (GRAMMS, MMAT) and formal ethical approval further strengthen the study’s rigor.

Several limitations must be acknowledged. The small and relatively homogeneous sample of 13 participants—many of whom held managerial roles and reported positive attitudes toward technology—limits generalizability and may reflect selection bias. Continuous involvement across multiple iterations may have influenced participants’ evaluations, as familiarity with successive prototypes could lead to more favorable perceptions of improvement. Social interaction with the research team may also have increased the likelihood of socially desirable responses.

The absence of a control group restricts the ability to obtain independent, neutral assessments of usability and perceived benefit. In addition, participation was limited to two facilities with prior experience in research projects, potentially underrepresenting settings or individuals who are more skeptical of or burdened by technology. Finally, evaluations were conducted in simulated environments rather than routine clinical practice, limiting conclusions about long-term usability, scalability, and impact on care outcomes. The study also focused on a limited set of technologies and did not include perspectives of care recipients. Accordingly, the findings should be interpreted as exploratory rather than confirmatory.

### Implications for Practice and Future Research

This study demonstrates that a unified, cross-device platform for robotic systems can be successfully developed through sustained end user involvement. Although the platform was initially designed to operate robotic technologies, needs articulated during the early requirements assessment led to the integration of VR content via the PICO 4 headset. This development illustrates the platform’s extensibility beyond robotics to other digital assistive technologies. Such flexibility is essential for future-oriented nursing environments, as it enables the centralized control of multiple technologies through a single interface while preserving usability and opportunities for personalization.

Nursing staff expressed willingness and motivation to use robotic technologies; however, successful implementation depends on intuitive usability, meaningful personalization, seamless workflow integration, and structured training. Quantitative improvements in autonomy and competence were reflected in design features that support familiar interaction patterns, centralized control of multiple technologies, and adaptation to individual care needs. Qualitative findings further emphasized the importance of personalization, transparent workflows, and integration with existing documentation systems for both acceptance and actual use. Collectively, these results suggest that technological rather than human factors represent the primary barriers to the integration of robotics into nursing care. Such barriers can be mitigated through collaborative design, iterative refinement, and close alignment with everyday care practices.

Future research should complement participatory development approaches with evaluations conducted by independent user groups who were not involved in the design process. This would allow assessment from a more neutral perspective and reduce potential biases associated with repeated exposure or social desirability. Expanding participant diversity, including staff with lower digital affinity, will further strengthen generalizability. Testing the platform in real-world care settings with larger and more heterogeneous samples and incorporating the perspectives of care recipients will be essential to evaluate long-term acceptance, effectiveness, and impact. Given its open architecture, the platform provides a promising foundation for integrating additional assistive technologies and supporting broader digital transformation in nursing care.

## Supplementary material

10.2196/84118 Multimedia Appendix 1 Statistical analysis.

10.2196/84118 Multimedia Appendix 2 Qualitative evaluation.

## Abbreviations

EduXBot: Educational Exploration Robot Application Platform
GRAMMS: Good Reporting of a Mixed Methods Study checklist
ISO: International Organization for Standardization
MMAT: Mixed Methods Appraisal Tool
SUS: System Usability Scale
TENS: Technology-based Experience of Need Satisfaction
TUI: Technology Usage Inventory
VR: virtual reality
